# BRAF-activated LncRNA functions as a tumor suppressor in papillary thyroid cancer

**DOI:** 10.18632/oncotarget.10825

**Published:** 2016-07-24

**Authors:** Tian Liao, Ning Qu, Rong-Liang Shi, Kai Guo, Ben Ma, Yi-Ming Cao, Jun Xiang, Zhong-Wu Lu, Yong-Xue Zhu, Duan-Shu Li, Qing-Hai Ji

**Affiliations:** ^1^ Department of Head and Neck Surgery, Fudan University Shanghai Cancer Center, Department of Oncology, Shanghai Medical College, Fudan University, Shanghai, 200032, China; ^2^ Department of General Surgery, Minhang Hospital, Fudan University, Shanghai, 201199, China

**Keywords:** papillary thyroid cancer, BRAF, lncRNA, proliferation, migration

## Abstract

Long non-coding RNAs (lncRNAs) participate in cancer cell tumorigenesis, cell cycle control, migration, proliferation, apoptosis, metastasis and drug resistance. The BRAF-activated non-coding RNA (BANCR) functions as both an oncogene and a tumor suppressor. Here, we investigated BANCR's role in papillary thyroid carcinoma (PTC) by assessing BANCR levels in PTC and matched normal thyroid epithelial tissues from 92 patients using qRT-PCR. We also used lentiviral vectors to establish PTC cell lines to investigate the effects of BANCR overexpression on cancer cell proliferation, apoptosis, migration and invasion. Our results indicate BANCR levels are lower in PTC tumor tissues than control tissues. Decreased BANCR levels correlate with tumor size, the presence of multifocal lesions and advanced PTC stage. BANCR overexpression reduced PTC cell proliferation and promoted apoptosis, which inhibited metastasis. It also inactivated ERK1/2 and p38, and this effect was enhanced by treatment with the MEK inhibitor U0126. Finally, BANCR overexpression dramatically inhibited tumor growth from PTC cells in xenograft mouse models. These results suggest BANCR inhibits tumorigenesis in PTC and that BANCR levels may be used as a novel prognostic marker.

## INTRODUCTION

The incidence of thyroid cancer, the most common endocrine malignancy, has increased by 3-fold over the last three decades. Approximately 80–85% of all thyroid cancers are papillary thyroid carcinoma (PTC) [1]. The 5-year survival rate of patients with PTC is over 95%; however, PTCs with more aggressive phenotypes can occasionally disseminate into distant tissues and lymph nodes or dedifferentiate into more lethal thyroid cancers [2]. Conventional management of PTC, including surgery, hormone therapy and radioactive iodine therapy are generally not curative in metastatic cases [1, 3]. Therefore, novel therapeutic approaches and individual treatment strategies are needed to treat patients with metastatic thyroid cancer [2]. For example, the genetic and epigenetic alterations associated with PTC pathogenesis may serve as novel prognostic markers or therapeutic targets.

Many long non-coding RNAs (lncRNAs; > 200 nucleotides in length) participate in cancer cell tumorigenesis, progression, proliferation, metastasis and apoptosis [4]. For example, the upregulation of the lncRNA HNF1A-AS1 promotes tumor proliferation and metastasis in lung adenocarcinoma by regulating the expression of cyclin D1, E-cadherin and β-catenin [5]. Also, the lncRNA CCAL regulates colorectal cancer progression by activating the Wnt/β-catenin signaling pathway and suppressing activator protein 2α [6]. PTCSC3, another lncRNA gene, confers PTC predisposition and promotes carcinogenesis through the S100A4 pathway [2]. Additionally, lncRNA-Hh promotes cancer stem cell generation by activating the hedgehog signaling pathway [7].

BRAF-activated lncRNA (BANCR) was first identified by Flockhart RJ *et al.* in 2012 via RNA sequencing. This molecule is a non-annotated, 693-bp-long transcript on chromosome 9 that reduces cell migration in melanoma [8]. BANCR has been reported to function as a tumor suppressor in lung cancer [9] while acting as an oncogene in various types of cancer [9–12]. Here, we carried out *in vitro* and *in vivo* studies to elucidate BANCR's role in PTC.

## RESULTS

### BANCR expression is downregulated in PTC tissue and cell lines

**Figure 1:**
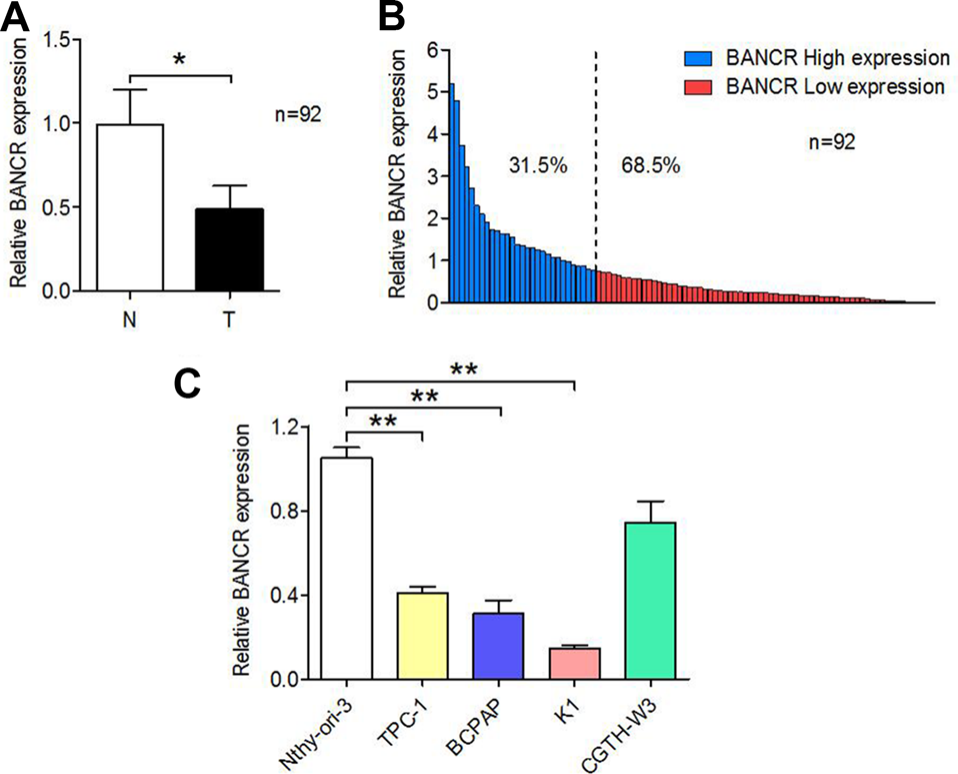
Relative BANCR expression levels in PTC tissues and cell lines (**A**) Relative levels of BANCR in PTC (T) and paired adjacent normal (*N*) tissues (*n* = 92) examined by qPCR and normalized to GAPDH mRNA expression. The results are presented as fold-changes in PTC tissues relative to normal tissues. (**B**) BANCR expression classified as high (31.5% of samples) or low (68.5% of samples). (**C**) Expression of BANCR in PTC cell lines (TPC-1, BCPAP, K1, and CGTH-W3) versus normal human thyroid epithelial cells (Nthy-ori 3-1). The results are presented as fold-changes in PTC cell lines relative to the Nthy-ori-3 cell line. (*P < 0.05, **P < 0.01).

To measure BANCR expression in PTC tissue, total RNA extracted from 92 paired human PTC samples and adjacent histologically normal tissues was assessed using SYBR Green qPCR analysis. As shown in Figure 1A–1B, BANCR expression was downregulated in 68.5% (63/92; *P* < 0.05) of the PTC tissue compared to the normal tissue.

Total RNA was also isolated from four PTC cell lines (TPC-1, K1, BCPAP, and CGTH-W3) and one normal human thyroid epithelial cell line (Nthy-ori 3-1) to evaluate BANCR expression. Compared to Nthy-ori-3 cells, BANCR expression was downregulated in TPC-1 (*P* = 0.0081), K1 (*P* = 0.0033) and BCPAP (*P* = 0.0119) cells but not in CGTH-W3 cells (*P* = 0.1129) (Figure 1C).

### BANCR overexpression inhibits PTC proliferation and induces apoptosis *in vitro* and *in vivo*

**Figure 2:**
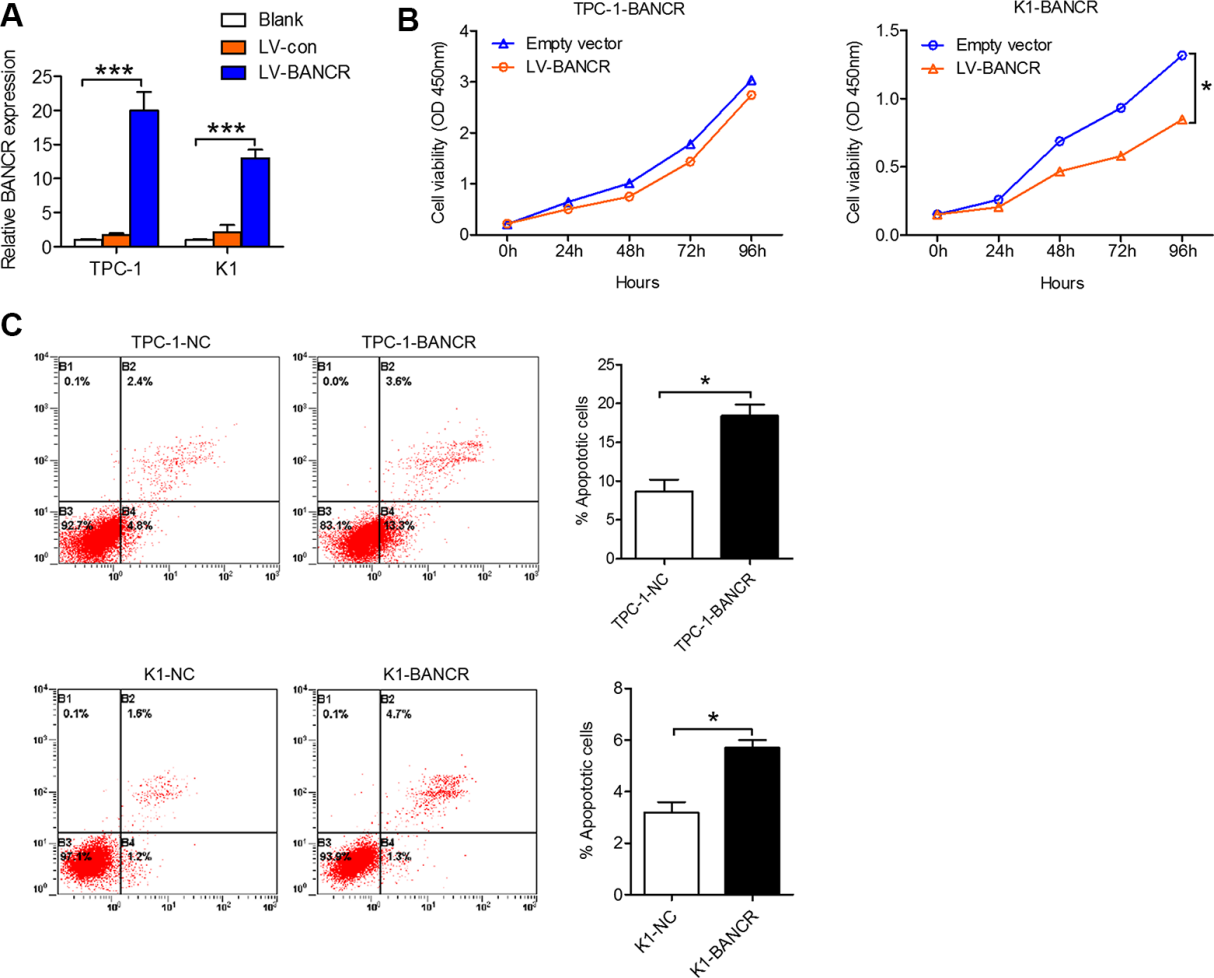
BANCR inhibits PTC cell proliferation and apoptosis *in vitro* (**A**) TPC-1 and K1 cells were transfected with lentivirus-BANCR and BANCR expression in the infected cells (TPC-1-BANCR and K1-BANCR) was confirmed by qPCR. (**B**) CCK-8 cell proliferation assays were performed to determine TPC-1-BANCR and K1-BANCR cell proliferation. The results are presented as the mean ± the standard deviation from three independent experiments. (**C)** Cell apoptosis was examined by flow cytometry. UL, necrotic cells; UR, terminal apoptotic cells; LR, early apoptotic cells. (*P < 0.05, ***P < 0.001).

To elucidate the role of BANCR in PTC cell proliferation and apoptosis, we established BANCR-overexpressing PTC cell models using the TPC-1 and K1 cell lines and lentivirus vectors. Increased BANCR expression in these infected PTC cell lines was confirmed using qPCR (Figure 2A). Proliferation of BANCR-overexpressing PTC cell lines TPC-1-BANCR and K1-BANCR was evaluated *in vitro* using a CCK-8 cell proliferation assay. Our results show that K1-BANCR proliferation is impaired compared to that of control cells (*P* < 0.05), while TPC-1-BANCR proliferation is unaffected (Figure 2B). Apoptosis analysis by flow cytometry showed that BANCR overexpression promotes apoptosis in both the TPC-1-BANCR and K1-BANCR cell lines compared to TPC-1-NC (*P* < 0.05) and K1-NC (*P* < 0.05) control cells (Figure 2C).

**Figure 3:**
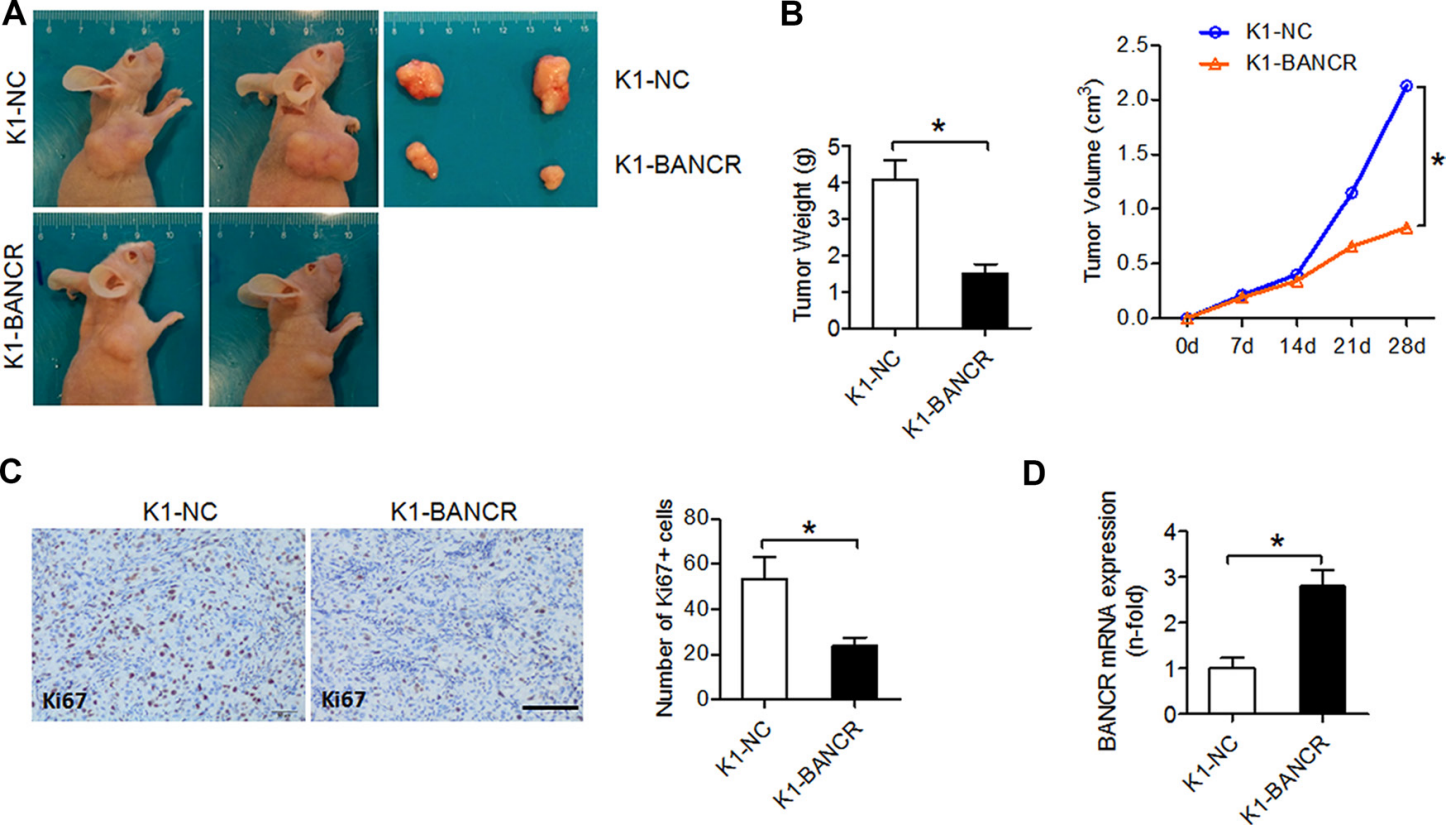
BANCR inhibits tumor growth *in vivo* BANCR-overexpressing K1-BANCR cells and K1-NC control cells were injected into BALB/c nude mice. (**A**) Two representative tumors from each group (*n* = 4) are shown. (**B**) Weight and volume comparison for tumors from K1-BANCR and K1-NC mice. (**C**) IHC analysis of proliferation marker Ki67 in formalin-fixed, paraffin-embedded tumors from K1-BANCR and K1-NC mice. The number of Ki67+ positive cells was counted using Image J software. Scale bar =100 μm. (**D**) BANCR levels in tumors from K1-BANCR and K1-NC mice determined by qPCR. The results are presented as fold-changes in the K1-BANCR group relative to the K1-NC group (*P < 0.05).

To investigate the effects of BANCR on PTC cell proliferation and apoptosis *in vivo*, we used subcutaneous xenograft mouse models engrafted with either K1-BANCR or control K1-NC cells. Average tumor weight and size were smaller in the K1-BANCR group than in the control group, indicating that tumor growth is inhibited in K1-BANCR mice (Figure 3A–3B). Furthermore, Ki67^+^ tumor cell number as measured by IHC is reduced (Figure 3C) and BANCR expression measured by qPCR is increased (Figure 3D) in the K1-BANCR group compared with the control group.

### BANCR overexpression suppresses PTC cell migration and invasion

**Figure 4:**
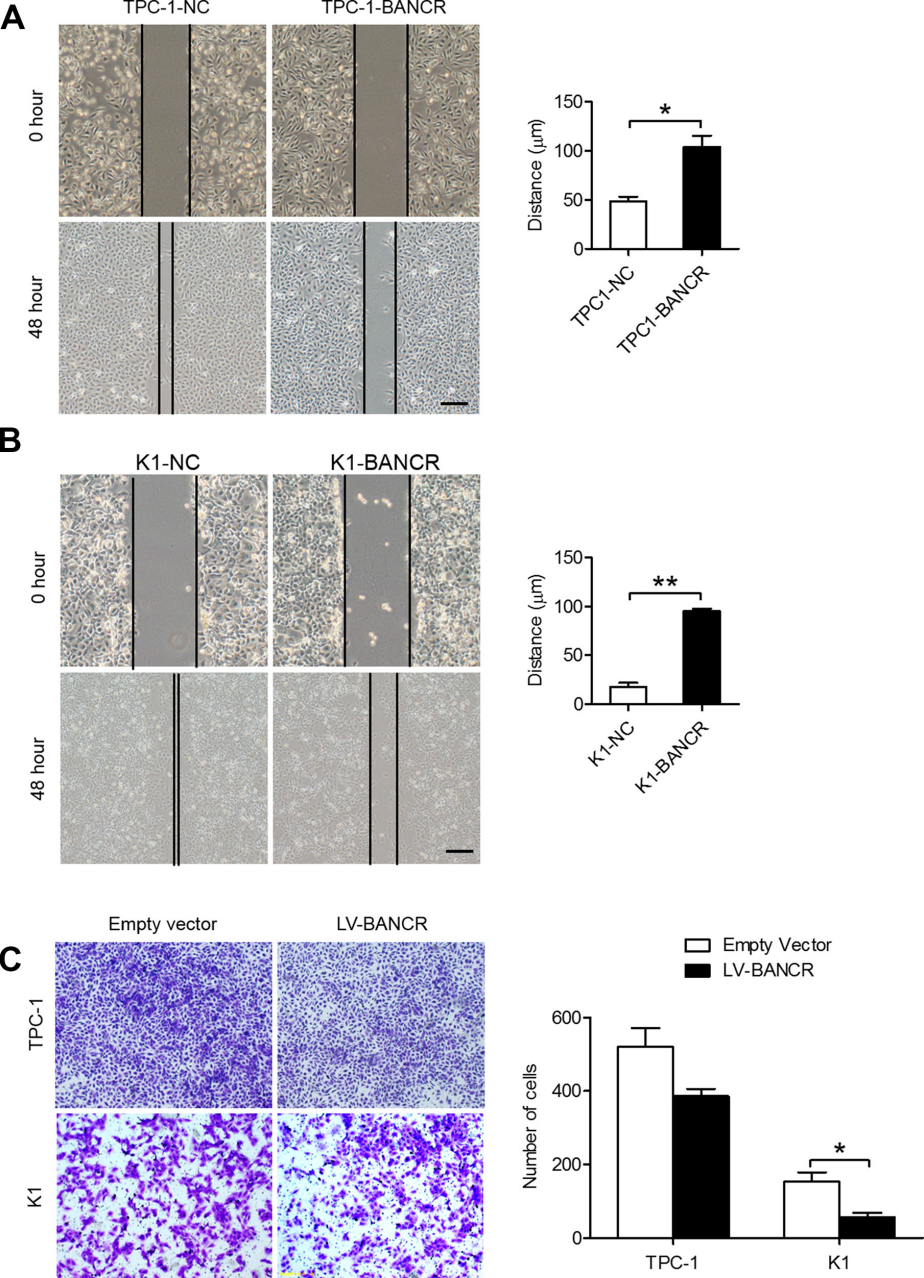
BANCR overexpression inhibits PTC cell migration and invasion *in vitro* (**A**–**B)** Wound-healing (scale bar = 10 μm) and (**C**) transwell assays showing the migratory abilities and invasive capacities of BANCR-overexpressing TPC-1-BANCR and K1-BANCR cells (*P < 0.05).

To investigate BANCR's role in PTC cell migration and invasion, we performed wound-healing and transwell invasion assays using TPC-1-BANCR and K1-BANCR cells. The wound-healing assay results revealed that BANCR overexpression slows down the closing rate of scratch wounds compared with that of control cells (*P* < 0.05) (Figure 4A–4B). The transwell invasion assay results showed that increased BANCR expression inhibits TPC-1-BANCR and K1-BANCR (*P* < 0.05) cell invasion compared with controls (Figure 4C).

### ERK1/2 and P38, but not AKT or JNK, are inactivated by BANCR upregulation

**Figure 5:**
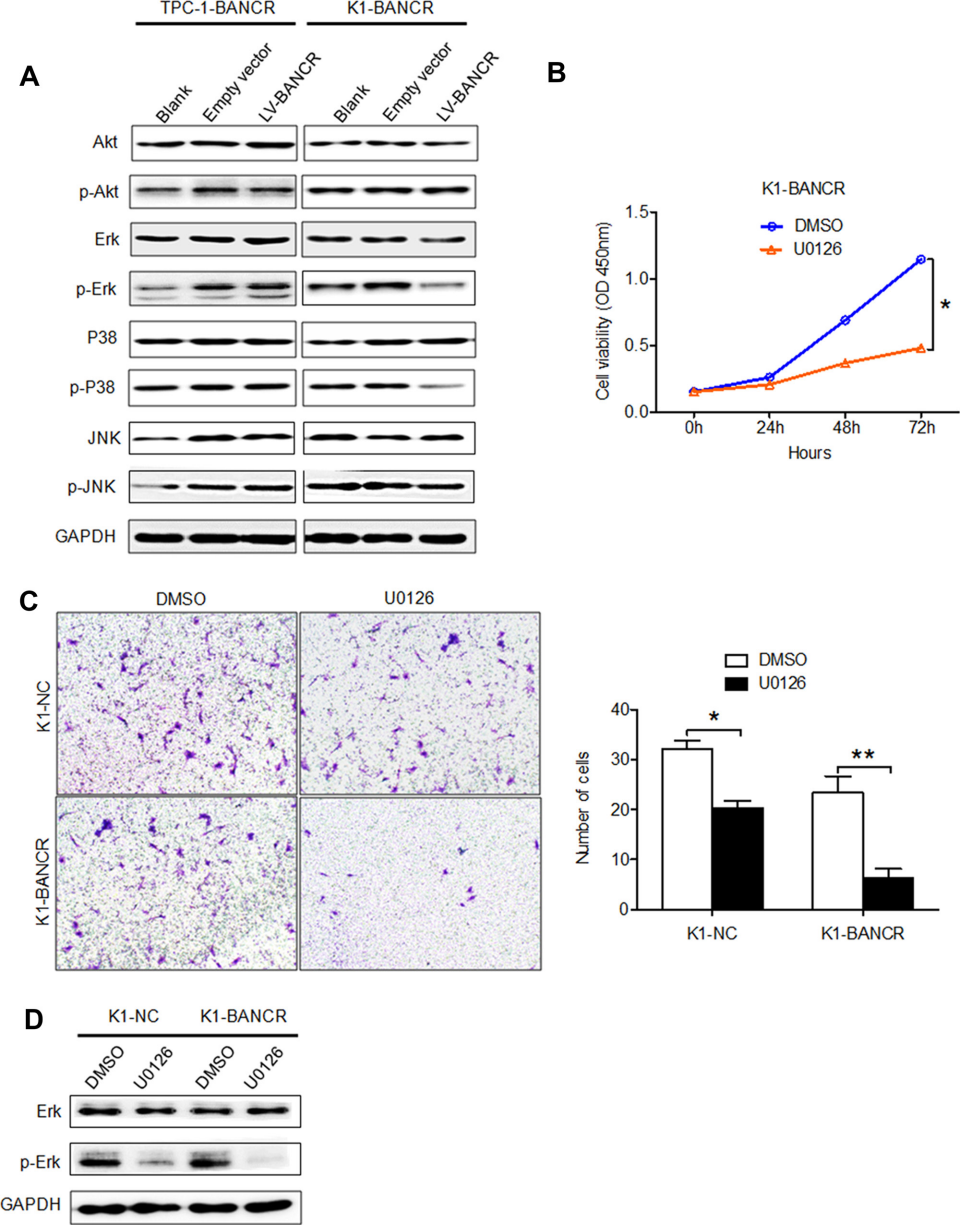
BANCR overexpression inactivates P38 and ERK, inhibiting PTC cell migration and invasion (**A**) Analysis of total and phosphorylated ERK, AKT, JNK and P38 expression in TPC-1-B and K1-BANCR cells. (**B**) CCK-8 proliferation and (**C**) Transwell assays showing the proliferative and invasive abilities of K1-BANCR cells treated with U0126. (**D**) Analysis of total and phosphorylated ERK expression in K1-BANCR cells treated with U0126. (*P < 0.05).

To investigate whether the ERK/MAPK signaling pathway is involved in the upregulation of BANCR observed in PTC cells, the expression levels of total and phosphorylated ERK, AKT, MEK, JNK and P38 were measured by western blot. As shown in Figure 5A, phosphorylated ERK and phosphorylated P38 were downregulated in K1-BANCR cells, but not in TPC-1-BANCR cells, while the total levels of these molecules (i.e., both phosphorylated and non-phosphorylated) did not change in either cell line. However, neither total nor phosphorylated AKT and JNK levels were affected in either cell line. In addition, treatment with the MEK inhibitor U0126 inhibited proliferation (*P* < 0.05) (Figure 5B) and impaired invasion (*P* < 0.05) (Figure 5C) in K1-BANCR cells relative to control K1-NC cells. Furthermore, levels of phosphorylated ERK were reduced in K1-BANCR cells following treatment with U0126 (Figure 5D).

### BANCR downregulation correlates with poor prognosis in PTC patients

To test whether BANCR correlates with prognosis in PTC patients, we measured the correlation between BANCR expression and several pathological parameters in PTC patients. Our results show that downregulation of BANCR expression correlates with tumor size (*P* < 0.05), the presence of multifocal lesions (*P* < 0.05) and advanced pathological stage (*P* < 0.05) in PTC tissues. However, BANCR expression did not correlate with other clinicopathologic parameters, such as age (*P* = 0.214), gender (*P* = 0.711), extrathyroidal extension (*P* = 0.406), or lymph node metastasis (*P* = 0.992) in PTC tissue compared with normal thyroid tissues (Table 1). These results suggest that low BANCR levels are associated with poor outcomes in PTC patients.

**Table 1:** The relationship between BANCR expression and clinicopathologic parameters in PTC patients

Clinicopathologic parameters	BANCR expression		*P*-value
	High (%)	Low (%)	
**Age (years)**			0.214
< 45	16 (55.2)	26 (41.3)	
≥ 45	13 (44.8)	37 (58.7)	
**Gender**			0.711
Male	6 (20.7)	11 (17.5)	
Female	23 (79.3)	52 (82.5)	
**Tumor Size (cm)**			0.046*
≤ 1	11 (37.9)	38 (60.3)	
> 1	18 (62.1)	25 (39.7)	
**Location of the primary tumors Solitary lesion**			0.579
Upper third	5 (17.2)	13 (22.4)	
Middle third	16 (55.2)	24 (41.4)	
Lower third	5 (17.2)	16 (27.6)	
Isthmus	3 (10.4)	5 (8.6)	
**Multifocal lesions**			0.038*
Positive	12 (41.4)	13 (20.6)	
Negative	17 (58.6)	50 (79.4)	
**Bilateral**			0.387
Positive	5 (17.2)	16 (25.4)	
Negative	24 (82.8)	47 (74.6)	
**Extrathyroidal extension**			0.406
Positive	2 (6.9)	8 (12.7)	
Negative	27 (93.1)	55 (87.3)	
**Lymph node metastasis**			0.992
Positive	14 (48.3)	30 (47.6)	
Negative	15 (51.7)	33 (52.4)	
**TNM Stage**			0.037*
I + II	25 (86.2)	41 (65.1)	
III, IV	4 (13.8)	22 (34.9)	

* *P* < 0.05, Chi-squared test *P*-value.

## DISCUSSION

Ectopic expression of BANCR shown in multiple cancer types may due to tumor heterogenicity; these findings motivated us to explore the effects of BANCR in PTC and to delineate the associated mechanisms. In the current study, we found that BANCR levels were decreased in 92 human PTC tissues compared to adjacent normal thyroid tissues. We also found that lower BANCR expression correlates with advanced tumor stage and size. We observed elevated BANCR levels in 86.2% early-stage PTC and 13.8% late-stage PTC tumors. Our results suggest that PTC tumors with high BANCR levels are more differentiated than those with low BANCR levels and that BANCR may function as a tumor suppressor in PTC.

We also investigated the role of BANCR in PTC cells utilizing BANCR-overexpressing TPC-1 and K1 cells as *in vitro* models. Our results show that BANCR overexpression inhibits cell proliferation, migration and invasion while inducing tumor cell apoptosis. In xenograft mouse models, K1 cells overexpressing BANCR exhibited lower tumor proliferation. Our *in vitro* and *in vivo* data show that BANCR acts as a tumor suppressor that inhibits tumor growth and metastasis, in line with the clinicopathological data. In a previous study [12], Zheng H et al. demonstrated that BANCR is upregulated in human PTC tissue and IHH4 cells and that this promotes cell growth and proliferation. The discrepancy with our results may stem from differences in the numbers of tissue samples tested (92 in our current study vs. 40 in Zheng H et al.) and in the BANCR expression patterns of different cell lines (we used TPC-1 and K1 cells while Zheng H et al. used IHH4 cells).

Activation of the MAPK signaling pathway promotes human cancer cell proliferation and survival, particularly in PTC [3, 13]. BRAF and RAS mutations activate the MAPK signaling pathway in thyroid cancer, inducing the expression of phosphorylated ERK1/2, JNK, and P38 kinase, amplifying oncogenicity [3]. BANCR is normally activated by BRAF; however, 30–60% of PTCs contain the BRAF^V600E^ mutation, which has been associated with higher mortality [14] and recurrence [15, 16] rates. BRAF-activated BANCR has been shown to promote cell migration and proliferation in melanoma [10] and lung carcinoma [17] via the MAPK signaling pathway. However, whether BANCR exerts MAPK-dependent effects in PTC remains unclear. Here we demonstrate that BANCR overexpression in the BRAF^V600E^-mutated PTC cell line K1 inactivates ERK1/2 and P38. Furthermore, this inactivation was blocked by the MAPK pathway selective inhibitor U0126, without altering BRAF's effects in wild-type TPC-1 cells. These results suggest interplay between the ERK/MAPK signaling pathway and BANCR in PTC, highlighting BANCR as a potential therapeutic target for the treatment of BRAF^V600E^-positive thyroid cancer.

In this study, we report that BRAF-activated BANCR may function as a tumor suppressor via the ERK/MAPK signaling pathway in PTC. However, our results might be biased towards early-stage PTC given the disparity between the number of early-stage (*n* = 66) vs. late-stage (*n* = 26) PTC patients in our sample. This difference might result from new diagnostic methods that allow detecting PTC earlier, yielding more patients with micro-papillary cancer (early stage; tumor size < 0.5 cm) than with papillary cancer (late stage; tumor size > 0.5 cm). Interestingly, the results from our cell proliferation and apoptosis assays varied for TPC-1 and K1 cells overexpressing BANCR. This might be attributed to the different BRAF status of these cells, warranting further investigations.

In conclusion, we demonstrated that BANCR induces proliferation, migration and invasiveness in BRAF-mutated PTC cells partially via the ERK/MAPK signaling pathway and that BANCR expression is downregulated in human PTC tissues. Therefore, our results suggest that BANCR may serve as a potential therapeutic target and that decreased BANCR levels could be used as a novel prognostic marker for PTC.

## MATERIALS AND METHODS

### Clinical specimen collection

A total of 92 pairs of PTC tumor and adjacent normal tissue were obtained from patients who underwent surgical resection at the Department of Head and Neck Surgery at Fudan University Shanghai Cancer Center between 2012 and 2015. The study was performed in accordance with the Helsinki Declaration and was approved by the Human Ethics Committee/Institutional Review Board of Fudan University Shanghai Cancer Center. Written informed consent was obtained from all 92 patients. The diagnosis of PTC was histopathologically confirmed, and no patient received preoperative treatment. The following clinicopathological characteristics were documented: age, gender, and PTC features, including tumor size, location, multifocal lesions, bilateral lesions, extrathyroidal extension, lymph node metastasis and TNM classification. The resected tissue samples were immediately snap-frozen in liquid nitrogen and stored at −80°C for further use.

### Cell culture and treatment

Four PTC cell lines (TPC-1, K1, BCPAP and CGTH-W3) and a normal human thyroid epithelial cell line (Nthy-ori 3-1) were used. Nthy-ori-3 and TPC-1 cell lines were provided by Dr. Haixia Guan. K1 was gifted by Dr. Zebing Liu. BCPAP cell lines were purchased from the Institute of Biochemistry and Cell Biology at the Chinese Academy of Sciences (Shanghai, China). The CGTH-W3 cell line was obtained from Dr. Jun Xiang. All cell lines were cultured in RPMI 1640 (GIBCO) supplemented with 10% fetal bovine serum (FBS; GIBCO), 100 U/ml penicillin and 100 mg/ml streptomycin (Invitrogen, Carlsbad, CA, USA) at 37°C in 5% CO_2_. For inhibitor treatment experiments, the cell lines were treated with either DMSO or 10 μM U0126 for 48 h.

### Total RNA extraction, reverse transcription and quantitative real-time PCR

Total RNA was extracted from tissues and cultured cells using TRIzol Reagent (Invitrogen) according to the manufacturer's instructions. A total of 1 μg of RNA was reverse-transcribed using a PrimeScript RT reagent kit (Takara, Dalian, China). For quantitative real-time PCR (qPCR), cDNA was amplified using SYBR Green Premix Ex Taq (Takara, Dalian, China) following the manufacturer's instructions. BANCR expression was normalized against GAPDH mRNA expression in three independent experiments. The following primers were used for qPCR: BANCR forward sequence, 5′-ACAGGACTCCATGGCAAACG-3′; reverse sequence, 5′-ATGAAGAAAGCCTGGTGCAGT-3′. The results were analyzed and calculated relative to cycle threshold values and then converted into fold changes.

### Generation of BANCR-overexpressing cell lines

Recombinant lentiviruses packing genomes encoding human full-length BANCR (LV-BANCR) and a negative control sequence (LV-NC) were purchased from GENECHEM (Shanghai, China). TPC-1 and K1 cells were infected with either LV-BANCR or LV-NC plus 5 μg/ml polybrene (GENECHEM, Shanghai, China). Infection efficiency was examined by qPCR 48 h after infection, and the cells were selected with 2 μg/ml puromycin to create stable overexpression cell lines. The infected TPC-1 and K1 cells were denoted as TPC-1-BANCR, K1-BANCR, TPC-1-NC and K1-NC in accordance with the lentiviral construct used in each. BANCR expression was confirmed by qPCR.

### CCK8 cell proliferation assay

Cell proliferation was determined using a cell counting kit-8 (CCK-8) assay according to the manufacturer's protocol. Briefly, cells were seeded into 96-well plates at 4 × 10^3^ cells/well. An aliquot of 10 μl CCK-8 solution was added to each well, and the plate was incubated for 4 h at 37°C. At the indicated time points, the absorbance at 450 nm was measured using a spectrophotometer. For each group, data from five wells were pooled.

### Analysis of apoptosis by flow cytometry

Cells were harvested, washed in phosphate-buffered saline (PBS), and stained with Annexin V-PE and 7-AAD in Annexin-binding buffer (BD Biosciences, Mississauga, ON) for 15 min at room temperature in the dark. As controls, cells were either not stained, stained with Annexin V-PE only or stained with 7-AAD only. Following the addition of 400 μl binding buffer, the cells were immediately analyzed by flow cytometry. Apoptotic cells were marked based on Annexin V expression (Annexin V+/7-AAD- and Annexin V+/7-AAD+), quantified and compared to the controls from each group. The experiment was performed in triplicate.

### Wound-healing assay

To evaluate cell migration, a wound-healing assay was performed. To accomplish this, 2 × 10^5^ cells/well were seeded into 6-well plates and cultured until the cells reached 90% confluence. Artificial wounds were created by scraping using a sterile 200 μl pipette tip. The streaked cells were then washed with PBS 3 times and cultured in medium containing 1% FBS for 48 h. Cell migration was observed at 0 h and 48 h by analyzing phase-contrast images that were captured using an inverted microscope. The distance between the two edges of a wound was calculated using Image J software.

### Transwell cell invasion assay

To perform a transwell invasion assay, 2 × 10^4^ cells were suspended in 100 μl serum-free medium and placed on the non-coated membranes of the upper chambers of a transwell plate insert (24-well insert, 8 μm pore size; BD Biosciences). Following this, 600 μl medium with 10% FBS was added into each lower chamber. After incubation at 37°C for 24 h, the cells remaining on the upper membranes were removed with a cotton swab. The migrated or invaded cells were fixed with 4% paraformaldehyde in PBS buffer, stained with 0.1% crystal violet, counted, and photographed under an inverted microscope over 5 different fields per filter. The experiments were independently repeated in triplicate.

### Western blotting

Cells were lysed in RIPA protein extraction reagent supplemented with a protease inhibitor cocktail (Roche, CA, USA) and phosphatase inhibitor cocktail (Roche). Protein concentration was measured using a bicinchoninic acid assay (BCA). Equal amounts of total protein lysate were separated by 10% SDS-PAGE and transferred onto PVDF membranes. After blocking in 5% non-fat milk, the membranes were probed with targeted primary antibodies overnight at 4°C and detected by incubating with specific secondary antibodies for 2 h at room temperature. ECL chromogenic substrate with HRP was used to visualize the protein bands, and the intensities of the bands were quantified using Image J software. GAPDH served as an internal loading control. Primary anti-human antibodies against total Akt, Erk1/2, P38, and JNK and phosphorylated-Akt, Erk1/2, P38, and JNK were purchased from Cell Signaling Technology (MA, USA).

### Tumour xenograft animal model

All animal experiments were performed based on a protocol that was approved by the Shanghai Experimental Animal Center (Chinese Academy of Sciences). Specific pathogen-free (SPF) female athymic nude mice between six and eight weeks of age were purchased from the Shanghai Laboratory Animal Center. After one week of being housed and fed in a designated animal room, each mouse was subcutaneously inoculated into the right scapula with either 1 × 10^7^ K1-BANCR cells or control K1-NC cells in 50% Matrigel (BD Biosciences). Tumor size and weight were monitored twice a week. At 25 days after inoculation, the mice were sacrificed to assess tumor size. Tumors from each mouse (*n* = 4) were randomly selected for immunohistochemical (IHC) analysis. Fresh frozen tumor sections were used for mRNA analysis.

### Immunohistochemistry

Formalin-fixed, paraffin-embedded xenograft tissue was used for IHC analysis. Briefly, 5 μm-thick paraffin sections were deparaffinized in xylene and rehydrated in a 100%, 95%, 75% ethanol gradient. Then, the tissue sections were incubated in 30% H_2_O_2_ for 30 min to quench endogenous peroxidase activity. After antigen retrieval in heated 10 mM citrate buffer for 10 min, the tissue sections were immunostained with mouse anti-human Ki-67 primary antibody overnight at 4°C. Horseradish peroxidase-conjugated mouse secondary antibody was added for 1 h at room temperature. A DAB substrate development system was used to detect protein expression. Images were obtained using an Olympus IX71 inverted microscope. Ki67+ cells were counted using Image J software.

### Statistical analysis

All statistical analyses were performed using GraphPad Prism 5.1. The results are presented as the mean ± the standard error of the mean. The statistical significance of inter-group differences was determined using the Student's *t*-test. Correlation analyses were assessed using the Pearson correlation and linear regression analyses.
